# Examining the impact of alerting visual stimuli on the effectiveness of practising surgical scrub technique in medical education

**DOI:** 10.1186/s12909-025-08479-8

**Published:** 2025-12-27

**Authors:** Erzsebet Vanyolos, Eszter Lidak, Mihalyne Boros, Karolina Kosa, Gabriella Gomori, Nikolett Orosz, Katalin Peto, Norbert Nemeth

**Affiliations:** 1 Department of Operative Techniques and Surgical Research, Faculty of Medicine, University of Debrecen, Moricz Zsigmond krt. 98, Debrecen, H-4032 Hungary; 2 Department of Behavioural Sciences, Faculty of Medicine, University of Debrecen, Debrecen, Hungary; 3 Department of Hospital Hygiene, University of Debrecen Clinical Centre, Debrecen, Hungary

**Keywords:** Surgical hand hygiene, Surgical scrub technique, Medical education, Visual stimuli, Healthcare-associated infections, Patient safety, Generation z learning, Semmelweis scanner, Fluorescein UV light, Infection prevention

## Abstract

**Introduction:**

Inadequate surgical scrubbing is a key risk factor for surgical site infections (SSIs). Visual documentation of errors during training enhances learning while the Semmelweis Scanner provides objective quality control. This study hypothesized that displaying graphic images of severe SSIs in the scrub room would improve students’ surgical scrub discipline and effectiveness. By serving as a stark visual reminder of the consequences of poor hand hygiene, this approach was expected to enhance adherence to proper scrubbing protocols, especially among Generation Z students, who respond well to multisensory learning strategies.

**Methods:**

The study involved 121 medical students in the “Basic surgical techniques” course during the 2nd semester of 2023/2024. The last phase of the surgical scrub was performed with hand disinfectant containing fluorescein. Participant identification utilized Radio Frequency Identification (RFID). Measurements were taken 4 times with Semmelweis Scanner device (HandInScan Zrt.), examining error localization and proportion relative to the total hand area.

Students were divided into two groups: Group A (n = 58) performed scrubs without visual stimuli, while Group B (n = 63) was exposed to SSI images during two of four sessions. Performance was categorized as low, medium, or high based on error rates. Group B completed a questionnaire evaluating the images’ impact and their motivation for proper hand hygiene.

**Results:**

Between measurements 2 and 3, Group B (with visual stimuli) showed a significant increase in high performers (24 to 42) and a reduced error rate (11.98 ± 5.12% to 4.77 ± 3.06%). Of Group B’s 63 students, 60.3% found the SSI images motivating, 34.9% were neutral, and 4.8% found them disturbing or distracting. All Group B students acknowledged hand hygiene’s critical role in infection prevention. Additionally, 44.4% recommended more frequent Semmelweis Scanner use, and 36.5% suggested additional visual tools to enhance scrubbing efficiency.

**Conclusions:**

Graphic SSI images as visual stimuli significantly improved surgical scrub techniques by boosting motivation and accelerating the learning curve. This method supports the prevention of healthcare-associated infections and enhances patient safety. Due to its effectiveness, the approach was integrated into the “Basic surgical techniques” course starting the following semester, with potential for broader application in medical education.

**Supplementary Information:**

The online version contains supplementary material available at 10.1186/s12909-025-08479-8.

## Introduction

Healthcare-associated infections (HAIs) play a major role in adverse health care outcomes contributing to increased morbidity, mortality, length of hospitalization, and financial burden [1]. The most common route of transmission of pathogens is through contact due to improper scrubbing before surgery, a principle first established by Ignaz Semmelweis in the 1840s. However, traditional visual inspection is often insufficient to guarantee proper disinfection. To ensure objective validation, digital technologies such as the Semmelweis Scanner have been introduced to identify missed areas and quantify hygiene quality. [2–5]. This can be achieved by teaching the protocol of surgical hand preparation [1] through various means that include demonstration, hands-on practice, and feedback [6]. Compliance must be monitored to ensure sustained adherence by staff to the protocol [7].

Teaching and practicing the technique of surgical scrubbing is essential in medical education that is optimally built on a solid theoretical basis followed by regular practice under the supervision of skilled teachers. The effectiveness of surgical scrubbing should be verified by examining the hands to identify errors and determine areas of deficiency, based on which suggestions can be made to correct the errors and draw attention to the possible consequences [8]. The easiest form of assessment is to demonstrate that the disinfectant covers all surfaces of the hand. Other, more elaborate methods include bacteriological sampling or testing hands in ultraviolet light with or without a scanner [9].

Challenges in surgical scrubbing are multifaceted, stemming from diverse factors such as ingrained routines, educational gaps, time pressure, knowledge attrition over time, clinical environmental influences, and the ‘master-disciple’ effect [3, 10–15]. Studies indicate that residents often deviate from protocols, shortening scrub times, inadequately rubbing areas, or skipping steps [10–14]. University-taught theoretical and practical knowledge fades rapidly without reinforcement, leading trainees to adopt suboptimal techniques from senior colleagues under high ward pressure [3, 14–16]. Regular feedback and video analysis have proven effective in sustaining correct techniques [17–20]. Possible solutions encompass continuous training, mentoring, standardised protocols, and awareness-raising via motivational tools.

Our department teaches the compulsory course “Basic surgical techniques” for 3rd year medical students that includes, among others, training in proper surgical scrubbing.

To increase the effectiveness of the procedure, we decided to introduce specific visual stimuli for students during practice. Using visual illustrations not only helps to better understand the importance of hand hygiene but it also makes staff more motivated to follow good practice. Good compliance and motivation can effectively reduce the risk of HAIs [17–21].

Visual illustrations have traditionally had great significance in medical education ever since the publication of the first “modern” medical book written by Andreas Vesalius and illustrated by drawings of a student of Titian [22].The use of visual illustrations has been based on the centuries-old observation that images are more likely to be remembered than words (called “picture superiority effect”). A scientific explanation for this effect emerged only in the 1980s in the dual coding theory of Paivio who proposed that the mental representations of words and pictures rest on distinct modes. Words are arbitrary codes of objects, events, or ideas that are serially processed and retain their separate identities even when interconnected. Nonverbal representations may be images, sounds, sensations or actions that are related to emotions, and since they are analogous (rather than arbitrary) to aspects of reality that they denote, they are easier to recall. Paivio offered the dual coding theory as a unifying framework for educational psychology [23].

Others also have shown that immediate feedback, together with the use of visual aids are effective in improving both performance and motivation [24].

In addition to the visual feedback provided by the Semmelweis Scanner, which displays errors using colour coding (green for properly disinfected areas, red for missed spots) and their percentage, thus enabling students to identify and correct specific deficiencies in real time, visual stimuli were also used in the form of photos of surgical site infections (SSI) placed at eye level above the sink during the scrubbing process. Another consideration for the use of visual stimuli was the fact that most of our current students are members of Generation Z who require a multisensory approach to education since they have grown up under the influence of digital devices such as smartphones, smart televisions, etc. They tend to multitask but have difficulty maintaining their attention for long periods. They prefer visual, graphic information over texts, and are used to immediate, continuous feed-back. A number of authors pointed out that a multisensory approach is more effective in this generation compared to previous ones [25–30].

Our aim was to investigate: (1) the impact of visual and quantitative feedback after practice, and (2) the impact of visual stimuli during practice on medical students’ effectiveness of surgical hand scrubbing skills.

## Methods

The study was conducted at the Department of Surgical Operative Techniques and Surgical Research, Faculty of Medicine, University of Debrecen, Hungary, with the participation of 121 third-year medical students, in the second semester of the 2023/2024 academic year, as part of the “Basic Surgical Techniques” course.

### Participants and educational programme

The 14-week compulsory course is embedded within Hungary’s 6-year one-tier medical education programme (integrated MD curriculum), which provides a seamless pathway from basic sciences to clinical training without intermediate degrees. As a preclinical module in the third year, it focuses on foundational surgical skills through weekly lectures and practical sessions under tutor supervision. Surgical scrubbing is introduced in week 4 (demonstration and asepsis lecture) and practiced repeatedly (weeks 4, 5, 7, 9, 10, 13, 14), transitioning from guided to independent execution.

Performance is assessed based on tutor feedback and midterm tests, while the final assessment also includes a practical and an oral exam. This structure promotes progressive skill development in an authentic setting, standardizing procedures for global comparability [2].

The survey included all students, who volunteered after Ethics Committee approval (DE RKEB/IKEB: 6274 − 2022). Informed consent ensured Helsinki Declaration compliance and data anonymization, enhancing ecological validity by reflecting real motivation. The sample’s homogeneity (similar knowledge level) minimized confounders and provided statistical power (G*Power; see 2.4).

**Fig. 1:**
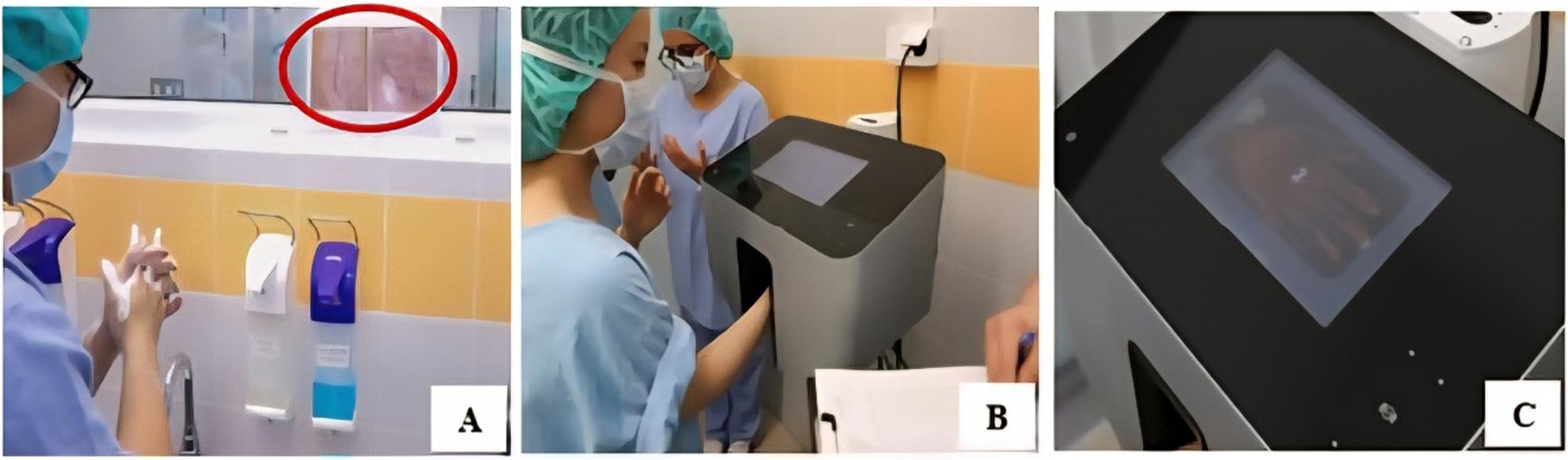
Steps of the assessment. **A**: During the whole scrub procedure photos of infected wounds were displayed in the scrub room (the circled area). **B**: Hand in scan device that detects the location of defects. Skin surfaces properly coated with fluorescein dye are clearly visible in UV light. **C**: The scanner analyses and shows the result of the scrub procedure, with green colour the properly treated and with red colour the improperly rubbed areas

Students were randomly divided into two groups: Group A (*n* = 58, no visual stimuli; control) and Group B (*n* = 63, photo-enhanced). Group B was exposed to SSI images during the entire scrub procedure (Fig. 1). Images were high-resolution (≥ 300 DPI) photographs of postoperative infected wounds, sourced from the Department of Surgery, University of Debrecen Clinical Centre, University of Debrecen. The photos showed typical signs of SSI on days 2–5 after surgery, including purulent discharge (yellow green), wound dehiscence, and tissue necrosis, recorded anonymously without identifying patient details (e.g., face, name, or unique marks). For this purpose, any image of an infected surgical wound (even from a textbook, free internet sources etc.) is appropriate. They were displayed in A4 size at eye level above sinks for optimal visibility during scrubbing. Patient consent was not required under GDPR as images were fully anonymised and non-identifiable; use was approved under the study Ethics Committee license (DE RKEB/IKEB: 6274 − 2022), with a data protection statement confirming no personal data processing.

Measurements occurred four times (weeks 5, 9, 13, 14) to capture the learning curve: week 5 (post-independence baseline), week 9 (routine acquisition), week 13 (error correction), week 14 (exam). This longitudinal design detects intervention effects, aligned with course progression (Table 1).

**Table 1:** Protocol of the application of visual stimuli during the practice weeks of hand scrubbing followed by evaluation (“Measurement”)

Group	Set-up	Week 5 (1st measurement)	Week 9 (2nd measurement)	Week 13 (3rd measurement)	Week 14 (4th measurement)
A	Practice condition	usual	usual	usual	usual
	Measurement focus area	RD, RP, LD, LP	RD, RP, LD, LP	RD, RP, LD, LP	RD, RP, LD, LP
B	Practice condition	photo-enhanced	photo-enhanced	photo-enhanced	photo-enhanced
	Measurement focus area	RD, RP, LD, LP	RD, RP, LD, LP	RD, RP, LD, LP	RD, RP, LD, LP

*RD* Dorsal surface of the right hand, *RP* Palmar surface of the right hand, *LD* Dorsal surface of the left hand, *LP* Palmar surface of the left

### Analysis of scrubbing effectiveness

Evaluation of the effectiveness of hand disinfection was carried out using Semmelweis Scanner (HandInScan Zrt.), described in detail by the inventors [31–32]. The students were identified individually for each measurement. After using fluorescein containing disinfectant for the last scrub, the contactless scanner automatically evaluated the adequacy of hand scrub and provided immediate and objective feedback in a time span of 30 s.

The device creates a visual image of the palmar and dorsal surface of the hand showing areas in green to show adequately treated parts and in red insufficiently rubbed areas. At the same time the programme provides quantitative information on the percentage of inadequately rubbed areas compared to the total hand surface. It also gives suggestions on which hand movements are most often missed and how to correct the gaps. By using RFID cards (Radio Frequency Identification) [33], the results can be loaded into a database, so adherence to the protocol can be monitored not only at the individual but at the institutional level as well.

Three categories were set up based on the percentage of performance achieved: low, medium and high performers. Above 95% students were considered high performers, between 80% and 94% medium performers and below 80% low performers.

### Questionnaire

After the final measurement in week 14, participants in group B (exposed to the photos) completed a questionnaire to assess the subjective impact of the photos on them. This included “closed” questions about their sex (male/female), dominant hand (right/left), the impact of the images on them (inspiration/motivation distraction no impact). They were also asked why hand hygiene is important to them and what methods they would suggest to further motivate them and teach the importance of hand hygiene more effectively (“open” questions). For the closed questions, the percentage of responses was summed up, and for the open questions, the ideas listed were collected, summing up the number and percentage of the identical ideas. The questionnaire, designed specifically for this study to assess the subjective impact of SSI images and student motivation for hand hygiene, is provided in Supplementary File 1.

### Statistical analysis

Statistical analysis was performed with all measurement results in both groups, examining the number and proportion of errors in relation to the total hand area, and the location of errors by hand surface and region. We also calculated the number of students who scrubbed incorrectly, along with sex differences.

SigmaStat Software 3.1.1.0 (Systat Software Inc., San Jose, CA, USA) was used to carry out the statistical analyses. Data are expressed as mean ± S.D. (standard deviation). The number of cases was estimated using the statistical program G*power. Differences between groups were analysed by t-test or the Mann–Whitney rank-sum test, differences between each measurements including both groups by paired t-test or Wilcoxon rank sum test, and the repeated measures ANOVA or Friedman’s test was used based on the results of the Kolmogorov–Smirnov normality test. A p-value of < 0.05 was considered statistically significant.

## Results

### Group performance analysis

**Fig. 2:**
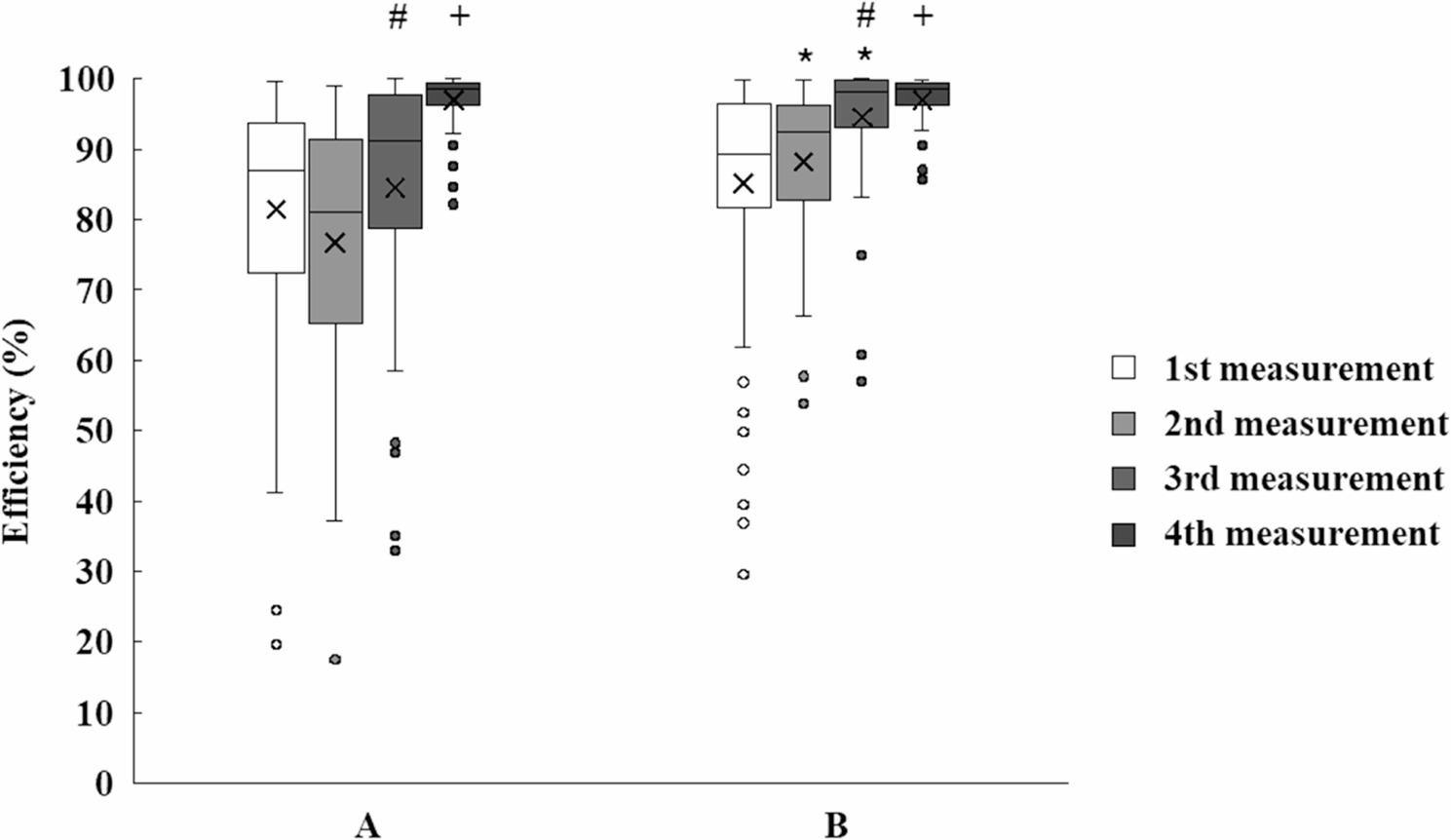
In efficiency significant differences were experienced between Group **A** and **B** at the 2^nd^ and 3^rd^ measurements. From 2^nd^ to 3^rd^ and from 3^rd^ to 4^th^ measurement significant differences (p<0.05) were found in both groups. Means ± S.D.; *p <0.05 vs. Group B # p <0.05 vs. 3^rd^ measurement + p <0.05 vs. 4th measurement

**Fig. 3:**
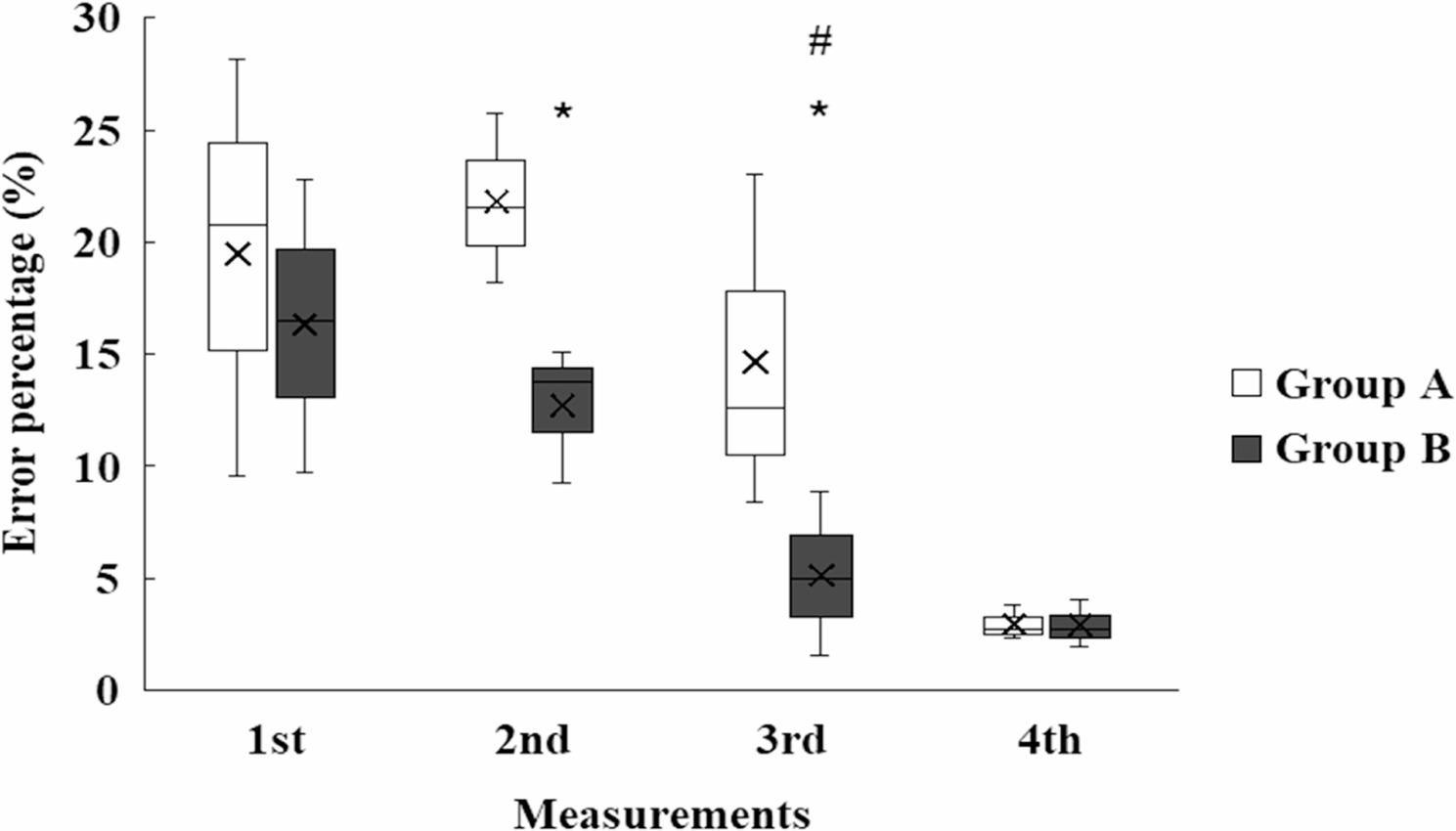
Students from Group B accomplished significantly less (p<0.05) error percentage than students from Group A. Means ± S.D.; *p<0.05 vs. Group B. # vs. 3rd measurement 2nd measurement *p=0.031; 3rd measurement *p=0.121; # p=0.051

**Fig. 4:**
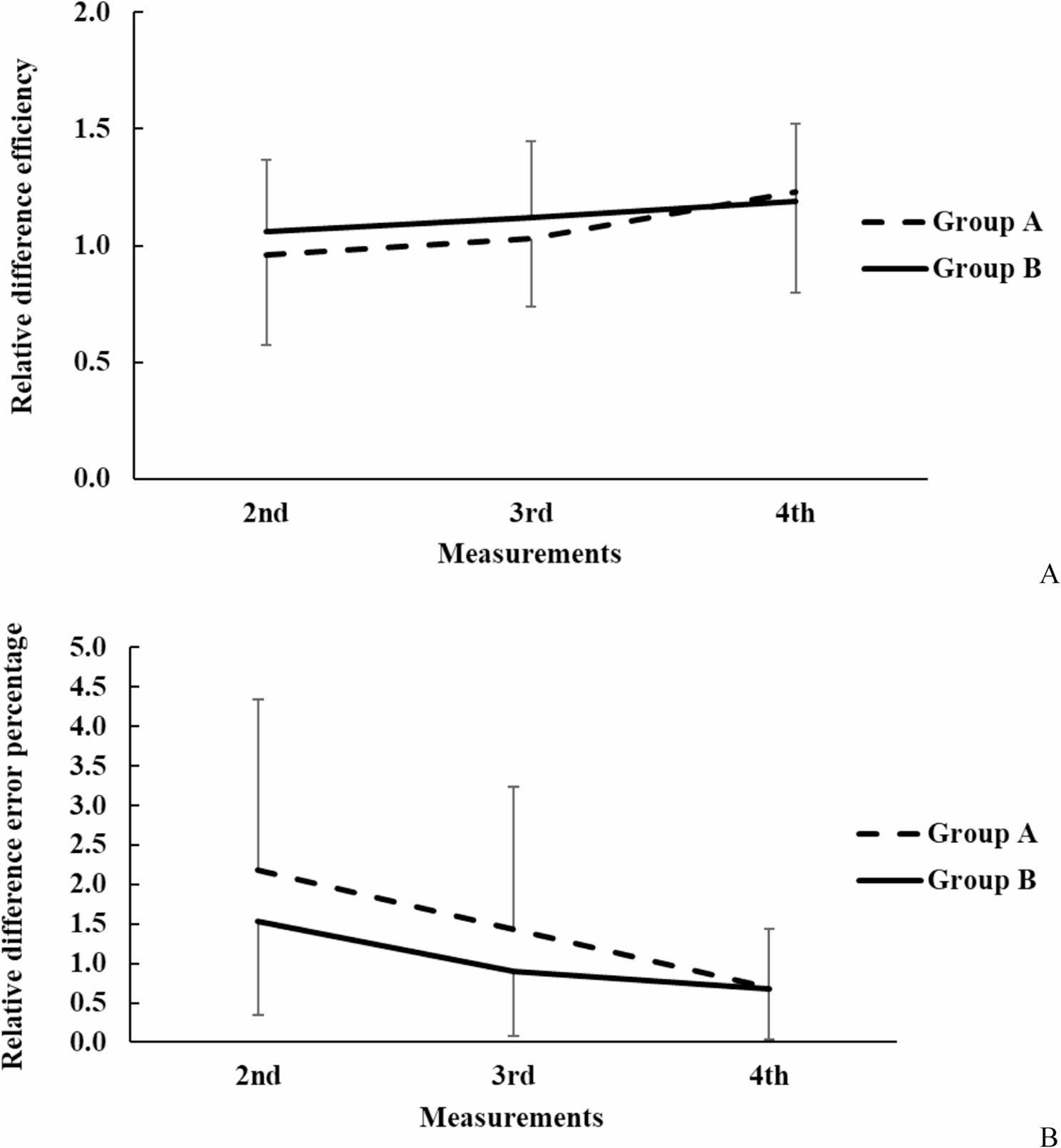
Relative changes of performance efficiency in Group **A** and **B** compared to the 1st measurement values. Visually depicts the relative improvement in surgical scrub efficiency for Group **A** and Group **B** compared to their baseline performance at the first measurement. It highlights the significant early advantage of visual stimuli in Group **B** (weeks 9 and 13) and the eventual convergence of performance by week 14, supporting the study’s findings on the effectiveness of SSI images in medical education

Of the 121 participants 41.3% (*n* = 50) were male and 58.7% (*n* = 71) were female; 92.5% (*n* = 112) were right-handed (41% male). Among the nine left-handed students, four were male. Between the 2nd and 3rd measurements, Group B (exposed to SSI images, *n* = 63) significantly “outperformed”, Group A (no visual stimuli, *n* = 58), with a reduction in procedural error rate from 11.98 ± 5.12% to 4.77 ± 3.06% (*p* < 0.001, paired t-test, Cohen’s d = 1.6) and an increase in high performers (≥ 95% disinfected area) from 24 to 42 (*p* < 0.001, chi-square test). Group A showed improvement by the 4th measurement, narrowing the performance gap (error rate: 5.12 ± 3.24%, *p* = 0.04 vs. Group B’s 4.65 ± 2.98%). Significant within-group improvements were observed from the 2nd to 3rd and 3rd to 4th measurements in both groups (*p* < 0.01, repeated measures ANOVA). Between-group differences were significant at the 2nd (*p* < 0.001, t-test) and 3rd measurements (*p* < 0.001, t-test), with Group B’s efficiency change (relative to the 1st measurement) being 10.4% higher at the 2nd measurement (1.06 ± 0.30 vs. 0.96 ± 0.38% points, *p* < 0.01) and 8.7% higher at the 3rd measurement (1.12 ± 0.32 vs. 1.03 ± 0.29, *p* < 0.01). By the 4th measurement, the efficiency gap was minimal (1.19 ± 0.33 vs. 1.23 ± 0.43, *p* = 0.62), indicating Group A’s catch-up through practice (Figs. 2, 3 and 4). 

### Localization of hand disinfection errors

**Fig. 5:**
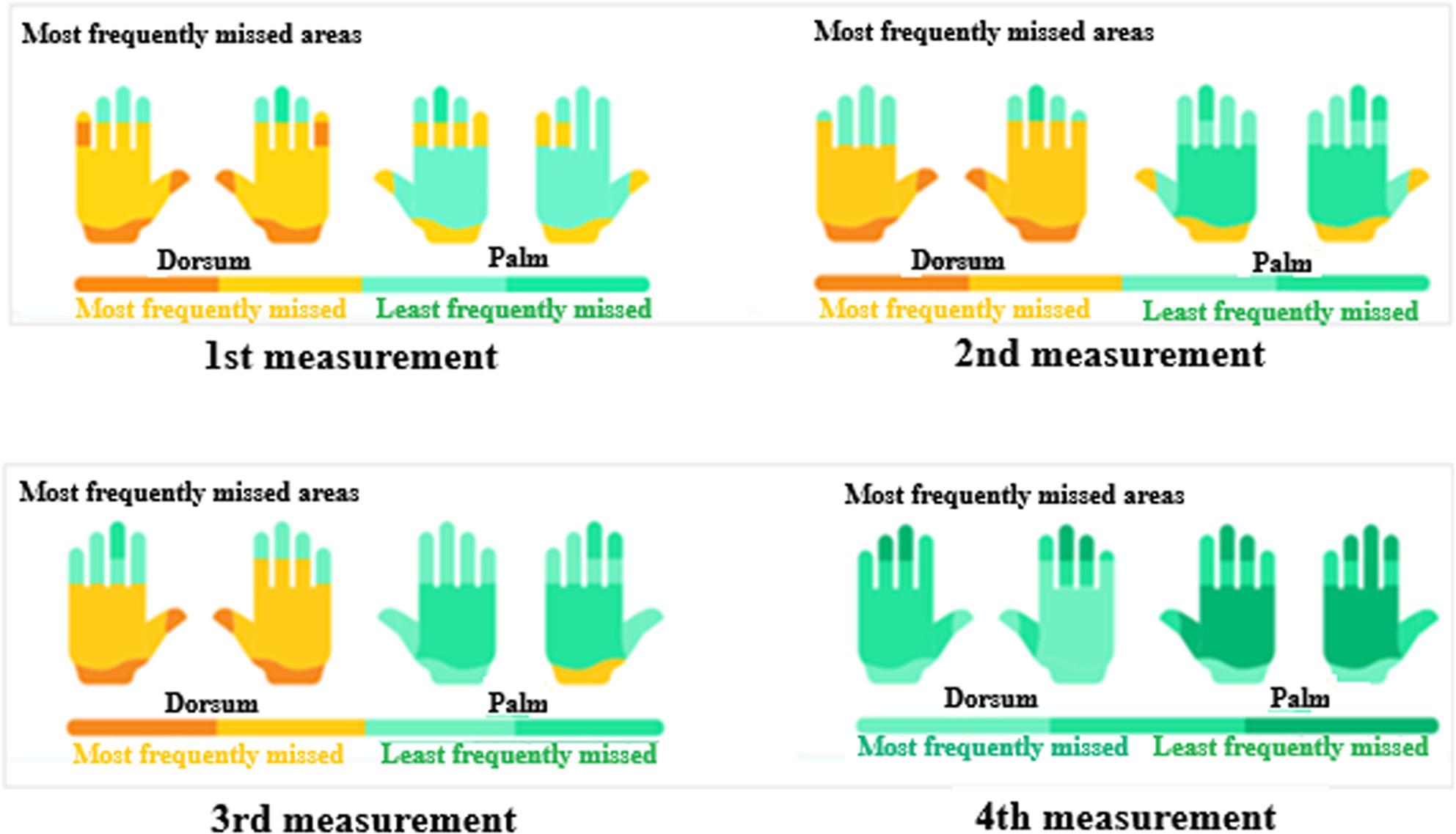
Most commonly missed areas during surgical hand rub across the 1^st^, 2^nd^, 3^rd^, and 4^th^ measurements, assessed by the Semmelweis Scanner. Yellow indicates dorsum (most frequently missed); green indicates palm (least frequently missed)

In the 1st measurement, 45% of the errors due to insufficient rubbing affected the back of the hand, followed by the thumb (30%). By the 2nd measurement, thumb errors had increased to 38%, and dorsum errors had decreased to 35%. In the 3rd measurement, thumb errors reduced to 25% and dorsum errors to 20%, with improved overall coverage (Table 2). By the 4th measurement, errors were minimal (10%), primarily around nail beds (7%) (Fig. 5). Table 2 shows the mean percentage of correctly disinfected areas (± S.D.) for dorsal and palmar surfaces of both hands across four measurements. Table 3 presents p-values for between-group comparisons, showing significant differences in palmar surface coverage at the 2nd (*p* < 0.001) and 3rd measurements (*p* < 0.01).

**Table 2:** The average percentage of missed spots areas (± S.D.) compared to the total hand surface area by measurement per hand surface (dorsal and palmar)

Focus area	Group	Measurement			
		1st	2nd	3rd	4th
RD	A	76.18 ± 23.02	58.67 ± 26.97	72.41 ± 28.69	94.61 ± 6.85
	B	78.12 ± 24.14	78.68 ± 20.73*	90.36 ± 15.52*	95.00 ± 7.06
LD	A	73.25 ± 26.64	66.66 ± 25.69	75.95 ± 26.14	95.73 ± 6.82
	B	76.84 ± 25.00	81.03 ± 20.09*	90.88 ± 15.89*	95.87 ± 6.86
RP	A	87.30 ± 17.57	90.32 ± 14.05	94.74 ± 11.40	98.75 ± 2.05
	B	90.33 ± 16.12	95.79 ± 5.89*	98.62 ± 2.69*	98.76 ± 1.77
LP	A	84.73 ± 19.98	91.11 ± 13.20	95.22 ± 10.12	98.71 ± 3.57
	B	90.32 ± 15.81	96.26 ± 4.52*	98.39 ± 3.33*	98.40 ± 2.24

*RD* Dorsal surface of the right hand, *RP* Palmar surface of the right hand, *LD* Dorsal surface of the left hand, *LP* Palmar surface of the left

Means ± S.D. *p < 0.05 vs. Group A

**Table 3:** The p-values of the comparisons between groups (Group A vs. B)

Measurements	RD	LD	RP	LP
1st	0.6536	0.4464	0.3255	0.0893
2nd	< 0.0001	0.0008	0.0053	0.0041
3rd	< 0.0001	0.0002	0.0102	0.0209
4th	0.7614	0.9182	0.9775	0.5707

*RD* Dorsal surface of the right hand, *RP* Palmar surface of the right hand, *LD* Dorsal surface of the left hand, *LP* Palmar surface of the left

### Sex and the dominant hand

No significant differences in scrubbing effectiveness were found between sexes (1st measurement: *p* = 0.257; 2nd: *p* = 0.933; 3rd: *p* = 0.286; 4th: *p* = 0.674, t-test). Right- and left-handed participants showed no significant differences (*p* > 0.05, Mann-Whitney test), though the small left-handed sample (*n* = 9) limited the statistical power, as noted in the Discussion.

### Performance categories (low/medium/high)

Table 4 shows the distribution of low (< 80%), medium (80–94%), and high (≥ 95%) performers. Group B’s high performer count increased from 24 to 42 between the 2nd and 3rd measurements (*p* < 0.001, chi-square test), with a 69% reduction in low performers (*p* < 0.01). Group A showed a slower increase in high performers (18 to 30, *p* < 0.01). No significant differences in medium performers were observed between groups or measurements (*p* > 0.05). Group B had significantly fewer low performers than Group A at the 2nd measurement (*p* < 0.05).

**Table 4:** Percentage of the high (≥ 95%), medium (80%-94%) and low (≤ 80%) performers

Performers	Group	Measurement			
		1st	2nd	3rd	4th
High performers [%]	A	18.97	13.79	36.21	77.59
	B	26.98	39.68	68.25	82.54
Medium performers [%]	A	51.72	39.66	39.65	22.41
	B	53.97	39.68	25.40	17.46
Low performers [%]	A	29.31	46.55	24.14	0
	B	19.05	20.64	6.35	0

### Questionnaire assessment

The questionnaire, developed for this study (Supplementary File 1), assessed Group B’s perceptions. Sex and handedness results are reported in 3.3. Of Group B’s 63 students, 60.3% (*n* = 38) found SSI images motivating, 34.9% (*n* = 22) were neutral, and 4.8% (*n* = 3) found them disturbing. For “Why is hand hygiene important?” responses included “infection prevention” (100%), “health protection” (21.66%), and “doctor-patient protection” (25.39%). Suggestions for improving hand hygiene training included “more scary photos” (96.82%), “more UV (ultraviolet) control” (92.06%), and “informational videos” (42.85%). Additionally, 44.4% (*n* = 28) recommended routine Semmelweis Scanner use.

## Discussion

Unlike our prior study using UV testing for efficacy monitoring in which we did not use extra stimuli [34], this investigation innovates by combining quantitative feedback with deterring SSI images during practice, with the aim of providing a novel motivational tool for Generation Z learners.

Our findings demonstrated that SSI images as visual stimuli significantly accelerated skill acquisition and reduced error rates in early sessions, aligning with the hypothesis that such deterrents enhance motivation beyond verbal instructions. Between measurements 2 and 3, Group B exhibited a marked improvement (error rate from 11.98% to 4.77%; high performers from 24 to 42), underscoring the role of confronting consequences in fostering compliance.

We hypothesized that photos of SSIs displayed during the training would have a greater impact on Generatation Z learners than verbal explanations. Our analysis revealed that the use of the photos was effective in teaching scrubbing, improving technical execution and increasing motivation.

The integration of visual stimuli (SSIs) into surgical scrub training significantly improved medical students’ adherence to hand hygiene protocols, reducing procedural errors and increasing the proportion of high performers, particularly among Generation Z students receptive to multimodal learning. This method improves the learning curve, bridges theoretical and practical skills, and contributes to the prevention of nosocomial infections and improves patient safety.

Mayer’s study has also demonstrated that visual stimuli (e.g. pictures, diagrams) can be useful in education, especially in health education [35]. They found that immediate feedback, and the use of visual aids can effectively improve both performance and motivation [36]. Various signs and posters can also increase effectiveness, both in terms of compliance and effectiveness in reducing (HAIs) [37]. Others highlighted that an electronic device (e.g. scanners) with instant visual feedback can also be effective in improving hand hygiene compliance [38, 39].

These results align with literature on visual aids in health education, where immediate feedback enhances performance [24]. Our intervention’s motivational boost mirrors deterrent images on cigarette packs, which initially increase quit intentions by 10–20% but fade over time due to habituation specifically, desensitization due to repetition (reduced emotional response from prolonged exposure, often within 6–12 months) [40, 41]. Similarly, hand hygiene campaigns yield early gains that wane without reinforcement [42], suggesting our short-term effects (e.g., 69% reduction in low performers in Group B) may require image rotation or integration with tools like the Semmelweis Scanner for sustainability.

Possible solutions involve leveraging motivation and raising awareness. The latter can be effectively improved by attention-grabbing visual stimuli, such as the display of photos of SSIs in the scrub room, as used in our research. According to the literature such stimuli can have an impact on the learning curve of medical students. The mechanism of action is partly due to ‘learning by understanding’.^34^

All these observations are consistent with our experience. The use of photos of SSIs was effective in teaching scrubbing, improved technical implementation and increased motivation. Despite individual differences, the use of this method may contribute to the prevention of HAIs and increases patient safety.

This study has some limitations: the short-term assessment over four sessions (weeks 5, 9, 13, 14) did not evaluate long-term retention of skills or their impact on clinical practice. Subjective questionnaire responses may be prone to social desirability bias, particularly for questions about motivation. Group B’s exposure to visual stimuli in two sessions may have limited the intervention’s full effect. Additionally, potential confounders such as prior knowledge, individual learning styles, or instructor variability were not controlled. The small number of left-handed participants (*n* = 9) reduced statistical power for handedness analyses. Future multi-centre studies with larger samples, longitudinal designs, and controls for confounding factors are needed to validate the intervention’s efficacy and sustainability. Interestingly, while 60.3% of students found visual stimuli particularly motivating, only a smaller proportion (44.4%) recommended more frequent use of the Semmelweis Scanner. This difference may indicate that Generation Z prefers emotionally appealing, non-judgmental feedback over performance-based evaluations, as scanners can evoke a "test situation," while images serve as passive reminders ("motivators") that students found less intrusive but effective.

## Conclusion

The use of visual stimuli (SSI images) during surgical scrub training enhanced the speed of skill acquisition and reduced error rates in third-year medical students, particularly in early training sessions. Combined with immediate feedback from the Semmelweis Scanner, this approach improved adherence to WHO hand hygiene protocols. While effective in our study, the method’s broader applicability requires validation in larger, multi-centre studies. The approach has been incorporated into the “Basic Surgical Techniques” course starting in the 2024/2025 academic year, with the potential to enhance infection prevention in medical education.

Future research should explore long-term sustainability and generational differences in response to visual-based pedagogical methods to refine targeted educational approaches and advance patient safety.

## Supplementary Material

Supplementary Material 1.

## Data Availability

The data presented in this study are available on request from the corresponding author. The data are not publicly available due to ethical constraints.
