# Supplementary material for: Examining the impact of alerting visual stimuli on the effectiveness of practising surgical scrub technique in medical education
Source: BMC Med Educ. 2025 Dec 27;26:300. doi: 10.1186/s12909-025-08479-8 (PMC12914892; doi:10.1186/s12909-025-08479-8)
Supplement: Supplementary file 1 — Supplementary Material 1. [file 12909_2025_8479_MOESM1_ESM.docx]

Supplementary File 1:

Instructions: This questionnaire is designed to evaluate your experience with surgical hand hygiene training in the "Basic Surgical Techniques" course. All responses are anonymous and will be used to assess learner engagement and improve the curriculum. Please answer all questions honestly. The questionnaire includes multiple-choice, open-ended, and demographic questions.

Section 1: Demographic Information

1. Gender:
   Please select one option:
   - Male
   - Female
2. Are you right- or left-handed?
   Please select one option:
   - Right
   - Left

Section 2: Hand Hygiene Training Evaluation

1. What did the sight of the image (e.g., SSI photo displayed on the wall during scrubbing) trigger?
   Please select one option:
   - a) Inspired
   - b) Distracted their attention
   - c) Had no effect on me
2. Why is hand hygiene important?
   Please provide a brief response (2–3 sentences):
3. How could students be motivated to practice more effective hand hygiene?
   Please provide a brief response (2–3 sentences):

Section 3: Additional Feedback

1. Notes, comments:
   Please provide any additional feedback or suggestions about the hand hygiene training or the use of ultraviolet fluorescence scanning in the course (optional):

Thank you for your participation!
